# Bleb leak revision with Tenon's autograft

**DOI:** 10.1016/j.ajoc.2024.102141

**Published:** 2024-08-12

**Authors:** Lauren E. Chen, Philina Yee, Andrew K. Smith, Austin Fox, Sameh Mosaed

**Affiliations:** Gavin Herbert Eye Institute, Ophthalmology Department, University of California, Irvine, School of Medicine. 850 Health Sciences Rd, Irvine, CA 92617, USA

## Abstract

This video demonstrates two surgical cases where autologous Tenon grafts are used to shut down leaking trabeculectomies. Variations in this technique with a rotational graft and a hinge graft are explored.

## Video related to this article

Below is a transcript of the audio for the corresponding Video

Video 1
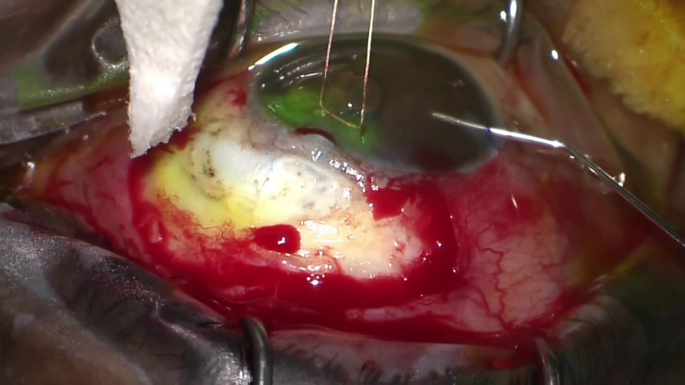

## 1 Intro

This video demonstrates a surgical technique for using a Tenon's autograft as a rotational or hinge flap to surgically manage a bleb leak, as performed by the glaucoma faculty at our institute. The following two cases are of eyes that had leaking trabeculectomies from melted conjunctiva and underlying sclera, where repair or reinforcement of the scleral defect was needed. The goal of this technique is to close and shut down the trabeculectomy, while providing structural support in areas of melted sclera or scleral flaps that do not adequately close through primary reapproximation.

## 2 Example 1: Rotational Autograft

This is an example of using a rotational Tenon's autograft to shut down a trabeculectomy that is both over filtering and leaking. This patient had a trabeculectomy 10 years prior with a provider outside of our institute and the intraocular pressure (IOP) was controlled off drops. However, upon routine follow up with us he was found to have worsening visual acuity, an IOP of zero, an ischemic bleb that was seidel positive, a shallow anterior chamber, and no choroidal effusions on fundus exam. Thus he was recommended to have a trabeculectomy revision with the goal shutting down the aqueous outflow through the scleral flap and eliminating conjunctival leak.

Here, we are operating on the right eye and sitting superiorly. The bleb is noted to be seidel positive and the ischemic conjunctiva is excised.

Balanced salt solution (BSS) is injected into the anterior chamber and flow is noted through the melted scleral flap. Primary closure by suturing the flap was not possible due to scleral melt seen here and additional tissue is needed for reinforcement.

A rotational Tenon's graft is created by dissecting a flap of superotemporal Tenon's off of the underlying sclera while leaving it attached nasally and rotating it 90° counterclockwise so it covers the site of the superonasal trab flap.

10-0 Prolene on a (blood vessel) BV needle is used to secure the anterior corners of the rotational Tenon's graft to the underlying sclera behind the limbus.

BSS is injected into the anterior chamber and no flow is noted, this accomplishes the goal of stopping all aqueous outflow through the trabeculectomy site.

The conjunctiva is then mobilized and advanced to the limbus, then closed with 10-0 vicryl suture.

Post-operatively, his IOP was initially controlled at 12–14 off eye drops. However, at post-op week 3 his IOP elevated to 22, for which he was started on latanoprost, dorzolamide-timolol, and brimonidine. At the patient's most recent post-op week 6 follow up, the IOP was acceptable at 12 on these four medications and the patient has not required additional IOP lowering surgery thus far.

## 3 Example 2: Hinge autograft

This next example uses a hinge Tenon's autograft to surgically manage a bleb leak. This patient had a trabeculectomy with mitomycin C (MMC) about 16 years ago with an ophthalmologist outside of our institute. However, upon routine follow up with us he was found to have worsening visual acuity to 20/40, an IOP of 10 off IOP lowering drops, a shallow anterior chamber, and an ischemic bleb that was seidel positive, without choroidal effusions on fundus exam. He was recommended to have a trabeculectomy revision with the goal of stopping the bleb leak. It was discussed that the planned technique for this revision will likely cause the trabectulectomy to fail and the IOP to rise, and if the IOP becomes uncontrolled despite maximum tolerated medications, subsequent IOP-lowering surgery can be performed if needed.

Here we are operating on the right eye and sitting superiorly. The ischemic conjunctiva was excised and an area of scleral melt is noted with aqueous flowing through. The focal application of MMC at high concentrations can lead to such scleral changes over time.

Due to scleral melt, primary closure was not possible and grafting material of some kind would be needed to cover the scleral defect. With the goal of shutting down the trabeculectomy, the fibrosed Tenon's posterior to the scleral flap was undermined to fashion a flap with an anterior hinge.

With a figure of 8 suture, 10-0 prolene on a BV needle is used to broadly secure the anterior edge of the hinged Tenon's graft to the underlying sclera, anterior to the melted trab flap site, right behind the limbus. The site was confirmed to be seidel negative, ensuring that the trabectulectomy site was shut down; and additional wing sutures were not needed in the corners of the hinged Tenon's graft.

When BSS is injected into the anterior chamber, there is no flow seen, accomplishing the goal of stopping all aqueous outflow through the trabeculectomy site.

Next the conjunctiva is reapproximated to the limbus with 10-0 vicryl in a running fashion.

Post-operatively the patient's IOP remained controlled. At post-op month 8 his IOP was 19 on dorzolamide-timolol and latanoprost, but he has not required additional IOP lowering surgery thus far.

## 4 Outro

The autologous Tenon's graft technique effectively addresses leaking trabeculectomies caused by melted conjunctiva and sclera that require the use of grafting material to reinforce the defective area. A Tenon's autograft has several advantages over scleral or corneal patch grafts. This technique utilizes the patient's own biocompatible tissue that is readily available, low profile, and expense-free.

Thank you for watching this video. We hope you will consider one of these techniques for bleb leak revision. Email us for any questions.

## CRediT authorship contribution statement

**Lauren E. Chen:** Writing – review & editing, Writing – original draft, Data curation. **Philina Yee:** Writing – review & editing, Data curation. **Andrew K. Smith:** Writing – review & editing, Formal analysis, Data curation. **Austin Fox:** Data curation. **Sameh Mosaed:** Writing – review & editing, Formal analysis, Data curation, Conceptualization.

## Declaration of competing interest

The authors declare that they have no known competing financial interests or personal relationships that could have appeared to influence the work reported in this paper.

The authors have no conflict of interest.

